# The Globally Rising Tide of Cosmetic Gynaecology: Are Providers Aware of the Ethical Aspects?

**DOI:** 10.7759/cureus.96327

**Published:** 2025-11-07

**Authors:** Shamail A Syed, Asila Anwar

**Affiliations:** 1 Obstetrics and Gynaecology, Maroof International Hospital, Islamabad, PAK; 2 General Practice, Claremont GP, Shrewsbury, GBR

**Keywords:** clitoral hood reduction, female genital cosmetic surgery, female genital surgery, high intensity focused ultrasound (hifu), hymenoplasty, labiaplasty, laser-therapy, medical ethics, platelet-rich plasma/ prp, vaginoplasty

## Abstract

The recent alarming rise in the popularity of female genital cosmetic surgeries seen globally has increased commercial opportunities in the aesthetic industry, but at the same time, medical professionals are faced with many ethical dilemmas revolving around the fact that often these procedures are not medically indicated. There is currently no robust scientific evidence available in regard to the safety and long-term outcomes of these procedures. Misleading marketing of these procedures has led to exploitation, especially of vulnerable populations like adolescents. There are also concerns that women might not be fully aware of the risks and potential long-term complications of a failed procedure requiring remedial surgeries, which can have irreversible implications on a woman's physical, mental and emotional health. The increase in demand is mostly driven by the desire to improve self-esteem and keep up with the unrealistic beauty standards set by society through social media platforms run by influencers and media celebrities. This has increasingly led to exploitation of young women's insecurities. There are only a few institutions worldwide offering formal training to professionals in the field of genital cosmetic procedures. In the developing world, there is a lack of regulatory authorities, which is particularly concerning when looking at the highly sensitive ethical aspects of this field. The procedures require expertise and should ideally be offered in accredited institutions by qualified professionals who offer well-researched procedures with realistic outcomes and high ethical standards.

The aim of this editorial is to urge gynaecologists, plastic surgeons and aestheticians offering these procedures specifically in the developing countries, to establish ethical guidelines and prioritize evidence-based care over their personal business interests. Educate women about the natural anatomical variations and empower women to make a fully informed decision based on an unbiased and honest disclosure of the limited evidence of long-term benefits and possible serious risks of these procedures.

## Editorial

We are living in an era of materialism where there is a growing fascination with looking perfect. The pursuit of flawlessness has extended to achieving perfect genitalia. This conquest has distorted the human perception of the normal anatomical appearance and has completely discredited natural diversity [1]. Cosmetic gynaecology has become the fastest growing subspecialty in medicine and surgery, and the urge in demand is multifactorial, influenced mainly by advertisements on social media and, to some extent, by the pornographic images and content readily available over the internet [1,2]. So, what women see on the web is now taken as the standard for normally appearing genitalia. Designer vulva, or a Barbie vulva, is a kind of labiaplasty procedure where the vulva is given a sleek look by reducing and tucking the labia minora under the folds of the labia majora. This and other such procedures that are being offered to women and marketed openly are setting unrealistic metrics which has left women feeling more anxious and insecure about their natural bodies. It is crucial to educate young women that just like our faces, no two vulvas can look the same. It is impossible to set anatomical standards as variations can exist amongst women of different ethnicities, ages, hormonal statuses and sexual history. A significant concern in this field is the emergence of industry-driven diagnosis, where certain medical devices are promoted as established treatments for correcting appearances and improving function of the female genitalia [2]. The surge in cosmgynaecological procedures is not a fleeting fad as the International Society of Aesthetic Surgery reported a 33.4% rise in cosmetic gynaecological surgeries between 2014 and 2018 [2]. Statistics from American Society of Aesthetic Plastic Surgery in 2018 showed an increase of labiaplasty procedures by 53% in comparison to the previous five years [3].

The American College of Obstetricians and Gynaecologists (ACOG), as well as the Royal College of Obstetricians and Gynaecologists (RCOG), defined female genital cosmetic surgery (FGCS) as procedures that are non-therapeutic in nature. These surgical or non-invasive procedures aim to change the look and functionality of a perfectly healthy external or internal genitalia [1]. The external genitalia includes the mons pubis, labia minora, labia majora, and clitoris, and the vagina is known as the internal genitalia. The types of procedures can broadly be divided based on the desired effects. It includes reduction and augmentation surgeries or procedures. Labiaplasty, perineoplasty, clitoral hood reduction, hymenoplasty, labia majora augmentation, vaginoplasty and G-spot amplification surgeries are some of the procedures being offered all over the world. Energy-based techniques such as radio frequency and laser therapy are also frequently being used in the field of cosmetic gynaecology [2]. There is limited evidence in literature on the effectiveness of all of these procedures. High-intensity focused ultrasound (HIFU) is a technique which causes focal induction of collagen and is used in vaginal rejuvenation. These procedures have rapidly gained popularity, allegedly aiming to enhance sexual function [4]. Energy-based devices, although they have shown some improvement in sexual function compared to placebo in some studies, have no optimal regimens or guidelines available for their standardised use [5]. The uncertainty about the long-term complications such as burns, scarring, altered sensation, dyspareunia, adhesions, vaginal stenosis and impact on mental wellbeing has not been studied yet [1]. In 2018, the US Food and Drug Administration (FDA) released a safety concern against the use of energy-based devices, including radio frequency and laser therapy, for cosmetic vaginal procedures or procedures intended to enhance sexual function [2]. 

While most of these procedures aim to enhance aesthetics, providers frequently make misleading claims, suggesting that some can also address functional concerns of women and improve sexual satisfaction in both partners. Female sexuality is very complex; it is an interplay of both anatomical and psychological factors [6]. The quality of evidence that has evaluated improvement in sexual function following such procedures is limited because of the complexity of sexual experiences and the lack of validated questionnaires for assessing vaginal tightness. Also, there is no data available regarding its long-term effects [6]. Vaginal rejuvenation and augmentation surgery are the commonest procedures being offered to improve sexual satisfaction. Advocates tout its potential by reducing vaginal calibre through surgeries like vaginoplasty or perineoplasty. Other techniques involve injecting platelet-rich plasma (PRP), adipose-derived stem cells, harvested from other parts of body, and hyaluronic acid fillers into the vagina walls, which thickens and reduces vaginal laxity, especially in women following vaginal births [4]. The limited data available show that these procedures have an inherent potential to cause new-onset dyspareunia due to the possibility of excessive narrowing of the canal. A rising interest has been observed recently in PRP or orgasm shots known as O-shot for vaginal rejuvenation. The injections aren't approved by the FDA, and the efficacy to date is debatable due to a lack of robust data [5].

Another notoriously famous procedure being frequently offered specifically in the Middle East and South Asian countries is hymenoplasty, which involves reconstruction of a torn hymenal tissue. Hymen is perceived as a sign of virginity in women, sought mainly for cultural and personal reasons. The appearance of a hymen is not a reliable indication of a woman not having had sexual intercourse, as it can naturally be absent in many girls [7]. The procedure has been declared illegal in the United Kingdom since 2022. The government of the UK has clearly banned hymenoplasty, declaring it as an offense and liable to a penalty under the Health and Care Act 2022 [8]. They have provided their health care practitioners with clear instructions on how to approach these situations where the decision for surgery is perceived to be coerced by societal pressures and fear of repercussions. There is a serious need for medical councils in the underdeveloped countries around the world to consider having similar guidelines in an effort to discourage such practices.

FGCS is not without risks and complications range from minor scarring and swelling to life-threatening conditions, re-hospitalisation and surgical intervention. The common risks are infection, bleeding, swelling, wound dehiscence, keloid formation, scarring, asymmetry and altered sensation leading to changes in sexual arousal [1]. The clitoris, composed of two highly sensitive erectile tissues lying in proximity to the labia minora, can easily be damaged during labiaplasty surgery, leading to lifelong sexual dysfunction, eventually having a negative impact on the quality of life. Rare complications include difficulty giving birth, urinary bladder control, need for remedial surgery and blood transfusion [1]. Furthermore, some women may experience dissatisfaction with the outcomes of these procedures, leading to lifelong regrets [1]. More so, there is no controlled evaluation data of short- and long-term clinical effectiveness and complications of FGCS in the literature. Most data available comes from case reports and retrospective studies, with significant variability in outcome measures [1,2]. Some of the barriers in collecting data include patient confidentiality issues and no long-term follow-up with the same provider, specifically in case of outcome dissatisfaction.

A rise in FGCS demand is seen amongst women of rapidly declining age groups, including teenagers younger than 14 years, which has raised a lot of ethical debate as emotional and psychological development is still ongoing and the societal beauty norms through social media can strongly shape their decision-making. The misconception that the labia should look the same as that seen in images on the internet, symmetrical and even, is the most common concern in adolescent girls. The British Association of Aesthetic and Plastic Surgeons (BAAPS) and the British Society for Pediatric and Adolescent Gynaecology (BritSPAG) strongly oppose and condemn any kind of aesthetic labiaplasty or genital surgery in minors for any non-medical reason [1]. Avoiding surgical interventions in adolescents unless thoroughly justified and treating these girls in a sensitive manner is the best approach, as both the external and internal genitalia continue to grow until adulthood. 

The issue of who should perform these surgeries is another debate; plastic surgeons may have an edge over gynaecologists to perform such surgeries with aesthetic precision, but gynaecologists may be a more suitable option for women with functional concerns [9]. There are many certification courses being offered around the world in the field of cosmetic gynaecology, but no credible platforms are offering standardised postgraduate resident training [8]. In a recent statement, the International Urogynecology Association has also raised concerns about these surgeries being performed by health professionals who aren't most suitable [9]. The role of dermatologists is also becoming more important as lasers are best known to them [3]. However, a dermatologist should be aware of what lies in the realm of dermatology and what needs to be referred to a gynaecologist [4,7].

In recent years, several gynaecological societies, including RCOG, ACOG, Royal Australian and New Zealand College of Obstetricians and Gynaecologists (RANZOG), the Society of Obstetricians and Gynaecologists (SOGC) and the International Federation of Obstetricians and Gynaecologists (FIGO) have raised concerns about FGCS and have strongly opposed the performance in the absence of valid scientific evidence [6]. According to a statement issued by FIGO, FGCS are ethically acceptable only if there is evidence available demonstrating the procedure being performed is safe and effective, the intended benefits outweigh possible risks and a fully informed consent is obtained from the individual with mental capacity to make a voluntary decision based on the information provided [2,10]. The statement emphasizes that these procedures are not medically necessary and often carry risks including infection, dissatisfaction with outcomes and changes in sexual function. Many physicians use their own websites and social media handles to publish educational materials regarding these procedures. The RCOG also recommends providing fully informed consent with clear instructions on risks and limited evidence of benefits to women, informing about the normal genital variations that can exist, offering psychological interventions for issues such as body dysmorphia if suspected, especially in adolescence, informing women and fully being aware themselves that they are providing procedures that lack a clear evidence base [2].

Women seeking genital cosmetic surgery in the clinics also provide an excellent opportunity for the clinicians to screen for underlying psychological issues such as body dysmorphic disorder (BDD), where women are obsessed with imagined defects in their appearance [2]. Screening for BDD by standardised screening tools and psychological therapy should be made a part of the consultation process in FGCS seekers, as it may give women a chance to process repressed thoughts and feelings which could help in identifying the underlying reasons for the request [6]. Evidence suggests that cosmetic surgery can sometimes lead to worsening of BDD symptoms [6]. Emotional agony may increase substantially in case of a failed procedure with disappointing outcomes [6]. For those women who do get diagnosed with disorders such as BDD, cognitive behaviour therapy is very effective if used as an adjunct or alternative to surgical interventions, according to some studies. While surgeries can give instant results to some, psychological therapy requires a lot of work and patience, but it can help with better outcomes and patient satisfaction rates when it comes to the kind of results a woman is looking for through these procedures [6].

The contemporary FGCS market is likely to grow in the future keeping the current dynamics in mind and therefore it is crucial for medical professionals to keep the core ethical principals in mind, respecting the woman's autonomy and acknowledging that the decision to proceed ultimately lies with her, provided that they fully know about the risks and alternative non-surgical treatments that are available [1,2]. Medical professionals should ensure that the woman is seeking a particular procedure for the right reason and not a result of coercion by a partner, societal pressure or to achieve unrealistic beauty standards that can significantly distort autonomous decision-making [2]. ​​​​In a study of 258 women undergoing FGCS, 32% of vaginoplasties were conducted to improve the partner's sexual pleasure [6]. A fully informed standardised comprehensive consent form should be developed by the gynaecological and plastic surgery societies covering limited evidence, potential risks, and alternative approaches [6]. Non-maleficence is the principle of doing no harm, hence, offering a surgical procedure with no proven benefits and possible physical and psychological harm should be avoided by all means [2]. The key to approaching women requesting FGCS is to empower and educate them to understand their body better, and help them make choices based on comprehensive knowledge about the natural body variations that can exist. Advertisements and discussions about these surgeries should be factual, evidence-based and impartial [2]. Offering cosmetic genital surgery merely for aesthetic reasons may also endorse the narrative of objectifying a woman's body, promoting unrealistic ideals of female genitalia and reinforcing the harmful societal attitudes towards female sexuality that can ultimately lead to making women feel inadequate. Ensuring that the woman seeking FGCS understands that cosmetic genital procedures are not medically indicated and the potential for physical harm and psychological distress far outweighs the desired benefits [2]. The providers should consider all the above-mentioned ethical issues associated with marketing and franchising FGCS. Cosmetic genital procedures are not being offered by national health services across the globe, unless it is for medical reasons like congenital ambiguous genitalia and most are being performed in the private sector [2], both in the developed and underdeveloped countries, raising questions about a key principle of medical ethics, which is equity and justice in health care. The high cost of these surgeries makes them accessible only to those who can afford them, further widening health disparities especially in developing countries where essential health care services are scarce to begin with [2].

A multidisciplinary team (MDT) approach should be offered to women considering FGCS, comprising gynaecologists, urologists, urogynaecologists, plastic surgeons, dermatologists and very importantly mental health professionals and, if relevant, legal counsellors for vulnerable populations such as adolescents and women with mental health disorders [4]. This would ensure adequate management in terms of avoiding litigation and patient dissatisfaction [1]. International regulatory bodies need to be set to standardize care, establish guidance on training and service accreditation in the field of FGCS. The procedures should be undertaken only by clinicians with appropriate expertise and accredited qualifications having enough case load to willingly audit and report their outcomes. We urge the international and national health care authorities and decision makers to take the initiative to make policies in order to monitor and regulate the cosmetic gynaecological procedures being performed and marketed over different social media platforms around the world, thus calling for medico-legal consequences related to practices not following the ethical core principles.
